# Using Human Primary Foreskin Fibroblasts to Study Cellular Damage and Mitochondrial Dysfunction

**DOI:** 10.1002/cptx.99

**Published:** 2020-11-17

**Authors:** Cristina A. Nadalutti, Samuel H. Wilson

**Affiliations:** ^1^ Genome Integrity and Structural Biology Laboratory National Institute of Environmental Health Sciences, NIH Research Triangle Park Raleigh North Carolina

**Keywords:** fibroblasts, cellular damage, mitochondria, mitophagy, transmission electron microscopy

## Abstract

Several cell lines of different origin are routinely used in research and drug development as important models to study human health and disease. Studying cells in culture represents an easy and convenient tool to approach complex biological questions, but the disadvantage is that they may not necessarily reflect what is effectively occurring in vivo. Human primary cells can help address this limitation, as they are isolated directly from human biological samples and can preserve the morphological and functional features of their tissue of origin. In addition, these can offer more relevant data and better solutions to investigators because they are not genetically manipulated. Human foreskin tissue discarded after surgery, for instance, represents a precious source for isolating such cells, including human foreskin fibroblasts (FSK), which are used in several areas of research and medicine. The overall health of cells is determined by the mitochondria. Alterations of cellular metabolism and cell death pathways depend, in part, on the number, size, distribution, and structure of mitochondria, and these can change under different cellular and pathological conditions. This highlights the need to develop accurate approaches to study mitochondria and evaluate their function. Here, we describe three easy, step‐by‐step protocols to study cellular viability and mitochondrial functionality in FSK. We describe how to use circumcision tissue obtained from the clinic to isolate FSK cells by mechanical and enzymatic disaggregation, how to use a cationic dye, crystal violet, which is retained by proliferating cells, to determine cell viability, and how to prepare samples to assess the metabolic status of cells by evaluating different mitochondrial parameters with transmission electron microscopy. We have successfully used the approaches outlined here to recapitulate physiological conditions in these cells in order to study the effects of increased intracellular levels of formaldehyde. © 2020 U.S. Government.

**Basic Protocol 1**: Isolation and maintenance of human primary foreskin fibroblasts (FSK)

**Basic Protocol 2**: Determination of cell viability by crystal violet staining

**Basic Protocol 3**: Transmission electron microscopy to study cellular damage and mitochondrial dysfunction

## INTRODUCTION

Circumcision is the most frequent surgical procedure worldwide and offers abundant material for different research applications (Somuncu, Tasli, Sisli, Somuncu, & Sahin, 2015). In this context, human foreskin tissue discarded after surgery represents a precious source for isolating human foreskin fibroblasts (FSK) for use in several areas of research and medicine (Oliveira et al., 2018). Fibroblasts are the most common cells of the connective tissue, and secrete collagen and other important components of the extracellular matrix (ECM), providing support to other cells, tissues, and organs in the body. Indeed, the capability of human fibroblasts to secrete proteins of the ECM is commonly exploited in research to maintain the undifferentiated and pluripotent state of stem cells (Unger et al., 2016). Fibroblasts have many important biological functions and play a central role in wound healing. These cells can proliferate and migrate to the site of injury, where they secrete collagen, glycoproteins, glycosaminoglycans, and fibers that support wound healing repair (Acharya et al., 2008). Consequently, fibroblasts represent an ideal in vivo cell model to study and investigate many cellular processes, for example, cell growth and differentiation, wound healing, fibrosis, and oxidative stress (Garrett, Baker Frost, & Feghali‐Bostwick, 2017; Nadalutti et al., 2020; Pizzino et al., 2017).

In eukaryotic cells, many metabolic processes, such as adenosine triphosphate synthesis, calcium regulation, and hormone biosynthesis are orchestrated by the mitochondria. Under physiological conditions, mitochondria regularly undergo fusion and fission, adjusting their morphology from granular to tubular forms, according to the metabolic demands of the cell (Youle & van der Bliek, 2012). Stress conditions and pathological disorders are known to alter mitochondrial morphology in terms of size, number, cristae distribution, and subcellular localization (Friedman & Nunnari, 2014). In addition, damaged or nonfunctional mitochondria are selectively removed by efficient quality control systems such as autophagy, which is central for preserving cellular homeostasis (Nunnari & Suomalainen, 2012). Autophagosomes can form around damaged mitochondria and become tagged with ubiquitin, which binds to specific autophagy receptors. This binding engages and recruits autophagy‐related proteins that isolate the cargo through the formation of double‐membrane structures. Once fully encapsulated, the autophagosomes fuse with lysosomes for content degradation (Dikic & Elazar, 2018). Altered autophagy has been linked to several pathological conditions (Dikic & Elazar, 2018; Jiang & Mizushima, 2014), and given the clinical importance of this process, it is highly relevant to have adequate techniques to measure it. Mitophagy represents a specific and selective form of autophagy, requiring methods for its specific evaluation, which would ultimately allow for the characterization of the contributions of both mitophagy and general autophagy.

**Figure 1:**
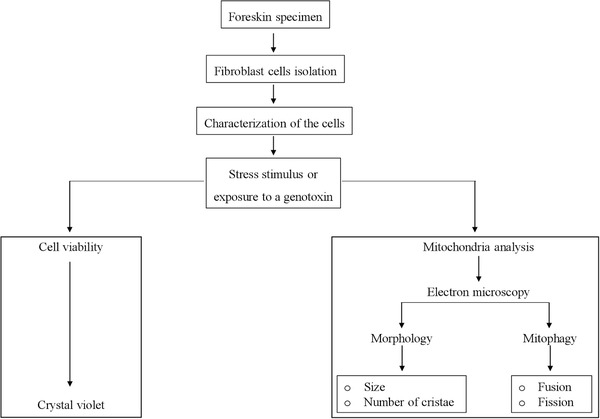
Flowchart of the experimental setup to study cellular damage and mitochondrial dysfunction using human primary foreskin fibroblasts (FSK). Isolation of FSK cells from human tissue (Basic Protocol 1) represents a valuable model to study, for instance, the effects of a genotoxin under physiological conditions. The research plan described in this article involves the isolation and characterization of the cells, the analysis of cell viability by crystal violet assay (Basic Protocol 2), and the evaluation of mitochondrial fitness by electron microscopy (Basic Protocol 3).

**Figure 2:**
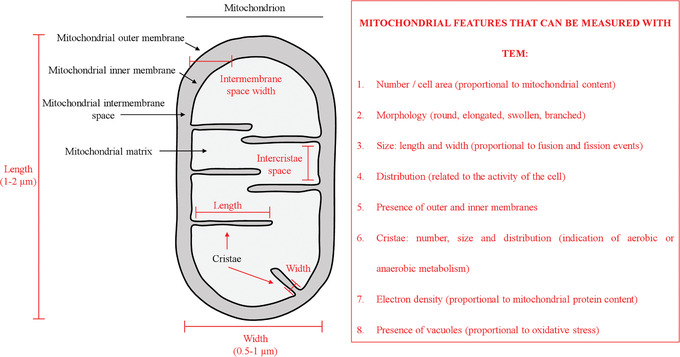
Diagram of the main parameters typically measured in mitochondria with transmission electron microscopy (TEM). Mitochondrial components are shown in black, and measurement parameters, in red.

In this article, we first describe a simple step‐by step protocol to isolate and culture FSK cells (Basic Protocol 1) to be used as an advantageous system to investigate cellular damage and mitochondrial dysfunction (Fig. 1). We next present a very easy method to assess FSK cell viability that employs crystal violet staining (Basic Protocol 2), which can be used to compare the impact of different growth conditions. Lastly, we introduce a step‐by‐step guide to preparing samples for using transmission electron microscopy (TEM) as a tool to monitor cellular behavior and mitochondrial fitness (see Fig. 2 and Basic Protocol 3). Altogether, these protocols will allow investigators to implement an alternative system to reduce the ethical and invasive techniques for acquiring cells of human origin, and support advancement in research.

## STRATEGIC PLANNING

The use of human‐derived material needs to satisfy all the requirements of the local applicable statutes and regulations. Consent from the donor is also an essential precondition. The experimental protocols described here, allowing the production of human FSK cells, respect guidelines and regulations of the National Institute of Environmental Health Sciences, National Institutes of Health. All biological experiments should be performed in a Biological Safety Level 1‐2 (BSL1‐2)–approved laboratory environment under sterile conditions in a laminar flow hood.

The foreskin specimen obtained from the clinic needs to be placed on ice immediately after surgery and kept in fibroblast complete culture medium (see Reagents and Solutions) during transportation. The isolation of the cells (Basic Protocol 1) should start immediately after arrival at the designated laboratory, under aseptic conditions. To prevent and limit contamination with other cell types, it is recommended to wash the tissue specimen several times before cell isolation (see Critical Parameters and Troubleshooting).

At the beginning of the culture process, it is recommended, in a laminar flow hood, to coat the petri dishes and the flasks for seeding and sub‐culturing with 0.1% gelatin (Sigma‐Aldrich, cat. no. C2124) for 1 hr at room temperature. The volume used for the coating should be sufficient to cover the surface area. After incubation, remove the coating solution and let the surface dry for 15‐20 min before plating the cells. Coated plates and flasks can be stored at 4°C for up to 1 week under sterile conditions. Let the petri dish or the flask adjust at room temperature for at least 1 hr prior to use.

In preparing the cells for transmission electron microscopy (TEM; Basic Protocol 3), every step of the procedure may affect the quality of the final micrographs. Therefore, cell processing should begin only after careful planning. Cell preparation for TEM can be divided into six major steps: fixation, washing, dehydration, infiltration, embedding, and cutting. The process begins with isolated human foreskin fibroblasts and ends with the cells preserved in a plastic resin. There are many ways to prepare cells for TEM. We adopted the protocol described here because it is simple and it efficiently preserves ultrastructure features of cells. Cutting resin‐embedded cells into extremely thin sections for viewing in TEM is a demanding technique that requires practice and patience. Sections must be very thin because the 50‐ to 125‐kV electrons of the standard electron microscope cannot pass through biological samples thicker than 150 nm. For best resolution, it is advisable that sections be in the range of 50 to 80 nm. This is roughly equivalent to splitting a 0.1‐mm‐thick human hair into 2000 slices along its long axis, or dividing a single red blood cell into 100 pieces. Thus, general prior knowledge of all of the equipment involved in the ultramicrotomy process is essential.

## Basic Protocol 1 ISOLATION AND MAINTENANCE OF HUMAN PRIMARY FORESKIN FIBROBLASTS (FSK CELLS)

**Figure 3:**
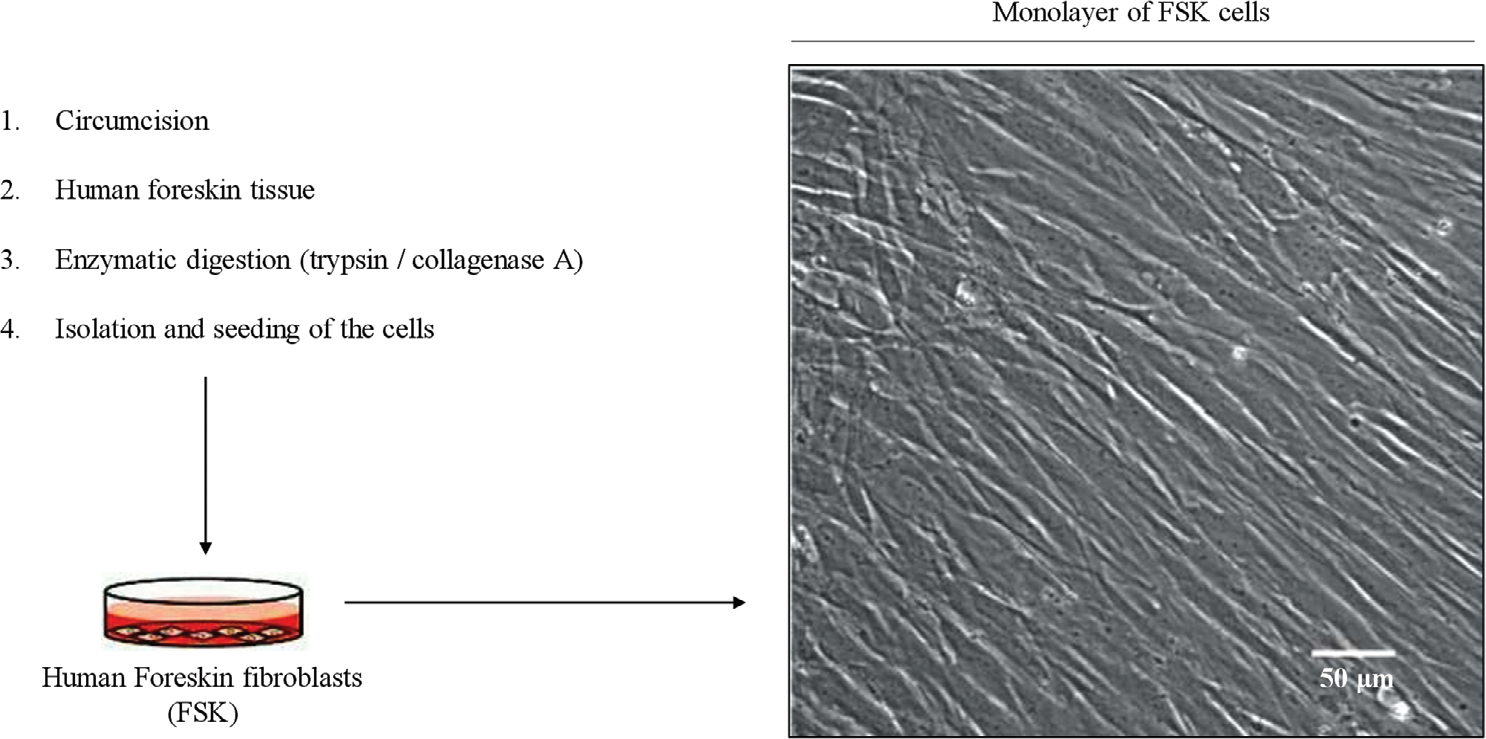
Confocal microscopy characterization of human primary foreskin fibroblasts (FSK). Human foreskin tissue obtained from circumcision is subjected to enzymatic digestion prior to isolation and seeding of the cells. The bright‐field image on the right (obtained using confocal microscopy) shows the characteristic morphology of human primary cell cultures of FSK cells in culture, at confluency. Scale bar = 50 µm.

Human primary cultures require the isolation of a specific cell type from tissues, for instance, from tissue discarded after surgery (Oliveira et al., 2018). Digestion of preputial skin allows one to efficiently isolate the cells of the foreskin, where fibroblasts are the most abundant cells of the dermis and the ones most commonly used for biomedical research. Even though the source of FSK cells may be limited because it is not possible to get extra material from the same donor, these cells can proliferate in the presence of serum, unlike some other cell types such as keratinocytes, which require additional culture supplementation for growth. Due to the lack of genetic manipulation, human primary cells are kept in culture only for a few passages, and are more sensitive than immortalized cells to small extracellular and intracellular changes. The cells start to grow out of the explant immediately after plating in a petri dish, and are easily maintained in the conditions described below until confluency (Fig. 3). Cells are inspected with an inverted microscope on a daily basis to observe normal growth.

FSK cells are cultured up to passage 6. To cryopreserve the cells, refer to Freshney (1987). Prior to use, the laminar hood should be subjected to UV light exposure (about 30 min) or cleaned with ethanol 70% (v/v) in water and kept aseptic during the whole procedure.

### Materials

Fresh human circumcision specimen obtained from a young healthy donor: size 2 × 2 cm^2^ (see Strategic Planning)
70% ethanol for cleaning laminar flow hood (see Strategic Planning)
Dulbecco's phosphate‐buffered saline (DPBS; Millipore, cat. no. D8537)
200 U/ml collagenase A (Sigma‐Aldrich, cat. no. C0130)
Complete fibroblast cell culture medium (FBM; see recipe)
0.05% (w/v) trypsin‐EDTA (Gibco, cat. no. T4049)

Laminar flow biosafety cabinet
37°C water bath
100‐ml beaker (Corning, cat. no. 1000‐100)
35‐ and 100‐mm plastic petri dishes (Corning, cat. no. CLS3294; Corning, cat. no. CLS3296), gelatin coated as described in Strategic Planning
Sterile forceps
Sterile tweezers
1.5‐ml sterile microcentrifuge tubes (Eppendorf or similar)
40‐μm sterile cell strainer (Corning, cat. no. 431750)
37°C humidified 5% carbon dioxide incubator
Eppendorf tabletop microcentrifuge
Inverted microscope
T‐75 cell culture flasks, vented cap (Sigma, cat. no. CLS3290), gelatin coated as described in Strategic Planning

Additional reagents and equipment for counting viable cells with a hemocytometer and trypan blue (see Current Protocols article: Phelan, 2007)

*NOTE*: All reagents should be purchased sterile or be filter sterilized using a 0.2‐μm filter.

### Cell isolation

1 Obtain circumcision specimen from the clinic and perform all of the following steps in an aseptic laminar flow hood.
2 Prewarm all solutions to 37°C.
3 Place the solutions and all the material required for the procedure under the hood.
4 Rinse the circumcision specimen with DPBS several times until blood is no longer visible.
5 Collect the waste in a beaker and follow your institution's biohazard disposal waste regulations.
6 Place the specimen on a 100‐mm petri dish and mince the tissue into 1‐2 mm pieces using sterile forceps.
7 Select a couple of pieces ∼1 mm^2^ in size and transfer them to a clean 1.5‐ml microcentrifuge tube using tweezers.
  The circumcision specimen that is not used can be stored up to indefinitely at −80°C in case you need to repeat the process due to unsuccessful isolation or low yields of cells.
8 Add 200 μl of collagenase A (200 U/ml). To allow digestion of the sample, incubate the tube at 37°C for 1 hr.
9 Gently flick the sample every 15 min.
10 Stop collagenase activity by adding 500 μl of complete FBM and filter with a sterile cell strainer to separate the cells from tissue fragments.
11 Microcentrifuge the filtered sample 15 min at 500 × *g*, room temperature,.
12 Discard the supernatant fraction. Wash by adding 1 ml DPBS, microcentrifuge 15 min at 500 × *g*, room temperature, and repeat steps 8‐11.
13 After centrifugation, resuspend the cell pellet in 1 ml of complete FBM.
14 Add 1 ml of fresh complete FBM to a 35‐mm coated petri dish (see Strategic Planning).
15 Count viable cells with a hemocytometer (see, e.g., Current Protocols article Phelan, 2007), and add 2 × 10^6^ of the resuspended cell pellet to a gelatin‐coated 35‐mm petri dish.
16 Place the petri dish in a humidified incubator at 37°C in an atmosphere of 5% CO_2_. After 24 hr, examine the cells using an inverted microscope.
  The cells will look round and will, with confluency, acquire the characteristic elongated shape (Fig. 3).

### Cell maintenance

17 After 24 hr, remove the medium and wash the cells with DPBS twice.
18 Add 2 ml of fresh FBM and let the cells grow at 37°C in an atmosphere of 5% CO_2_ for 48 hr.
19 After 2 days, check cell density with the inverted microscope.
20 If the cells are not confluent (70%‐80%), repeat steps 18 and 19 and let the cells grow for other 2 days.
21 When the cells are 70%‐80% confluent, proceed to subculturing into 100‐mm petri dishes.

### Sub‐culturing of cells

22 Warm up the reagents (except 0.05% trypsin‐EDTA) to 37°C at least 30 min before starting the procedure.
23 Remove the medium from the 100‐mm petri dish (from step 21) and wash the cells with 10 ml DPBS at least twice.
24 Add 500 μl of 0.05% trypsin‐EDTA and incubate at 37°C in an atmosphere of 5% CO_2_ for 3‐5 min to dislodge the cells.
25 Stop the reaction by adding 500 μl of complete FBM.
26 Transfer the cells to a fresh 1.5‐ml microcentrifuge tube and spin down the cells by centrifugation for 5 min at 500 × *g*, room temperature.
27 Discard the supernatant, add 1 ml of complete FBM, and resuspend the cell pellet.
28 Count viable cells with a hemocytometer (see Current Protocols article: Phelan, 2007) and plate 2 × 10^6^ cells in a 75‐cm^2^ gelatin‐coated flask with vented cap, with 25 ml complete FBM.
29 Incubate at 37°C in an atmosphere of 5% CO_2_ and change the medium every 3 days. Split the cells at 70%‐80% confluency and keep them in culture only up to passage number 6.

## Basic Protocol 2 DETERMINATION OF CELL VIABILITY BY CRYSTAL VIOLET STAINING

**Figure 4:**
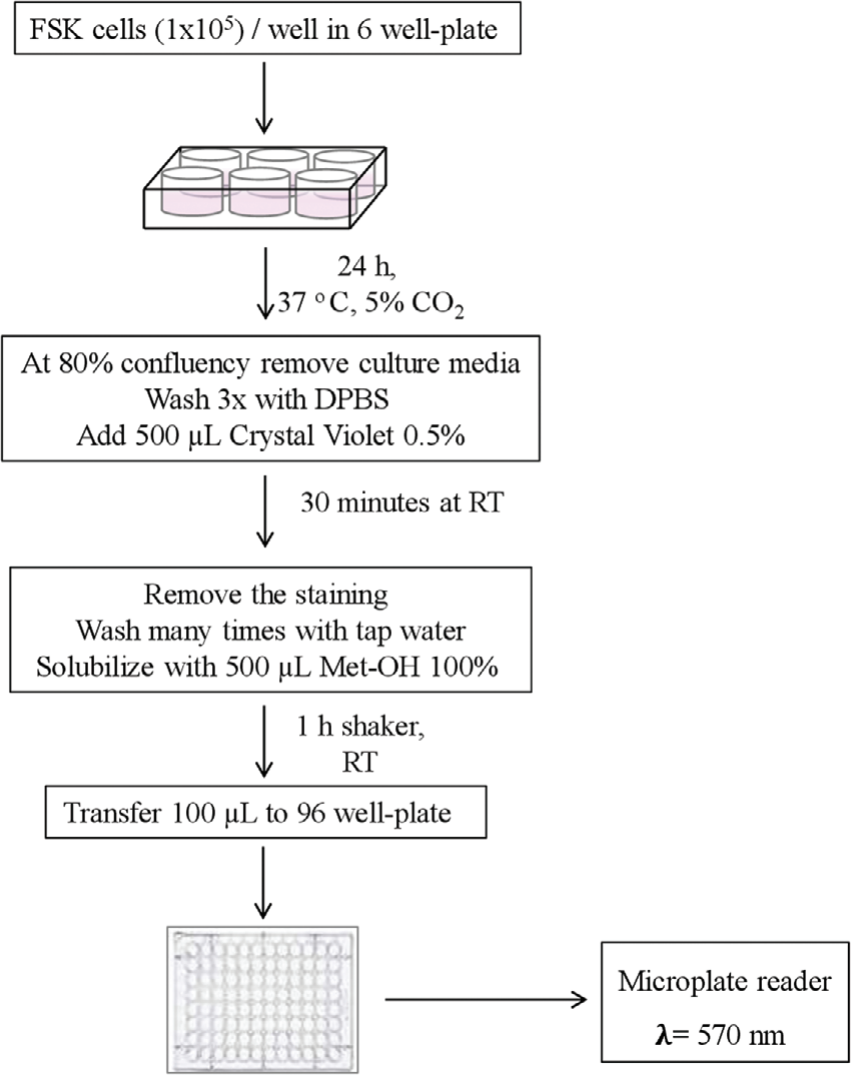
Flowchart depicting our strategy for measuring human primary foreskin fibroblasts (FSK) cell viability using crystal violet staining (Basic Protocol 2). DPBS = Dulbecco's phosphate‐buffered saline; RT = room temperature 25°C.

**Figure 5:**
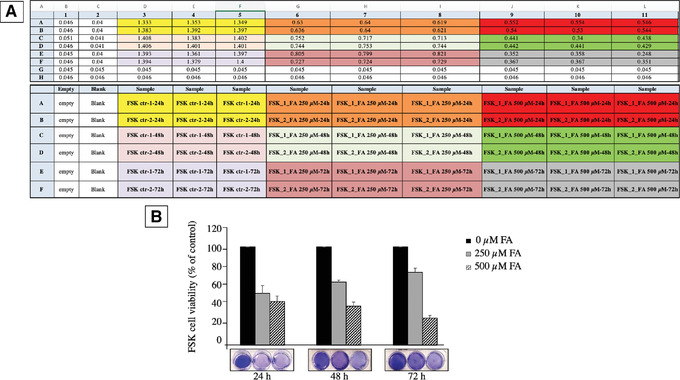
Example data from a crystal violet staining assay to evaluate formaldehyde (FA) cytotoxicity in human primary fibroblasts (FSK). (**A**) The top chart shows representative OD values obtained with a spectrophotometer at λ= 570 nm after crystal violet staining. The chart below depicts the experimental design. Blank corresponds to 100% methanol. The readings for methanol and empty wells should be close to zero. (**B**) FSK cell viability without and in the presence of 250 and 500 μM FA for 24, 48, and 72 hr. The cells showed different sensitivity to 250 μM FA and 500 μM FA treatments. Treatment of the cells with 250 μM FA was less harmful than exposure to 500 μM FA, which was highly cytotoxic at all the time points considered. Quantification of the OD values shown in A and expressed as percentage of control cells without any treatment. Average values were calculated from *n* = 6 (technical replicates) in each study group. All experiments were performed with different cell batches and repeated at least three times. The fluorescence intensity is taken as proportional to the number of viable cells. Data are presented as mean ± S.E.M.

We introduce a simple method based on crystal violet staining to evaluate FSK cell viability (Figs. 4, 5). This cationic dye works as an intercalating agent that enables the quantification of DNA, which is proportional to the number of cells in the culture, and the dye is easily sequestered by viable cells (Feoktistova, Geserick, & Leverkus, 2016). FSK cells form a monolayer when they become confluent. During cell death, adherent cells detach from culture plates and are washed away (and thus, the signal is reduced). In this context, crystal violet can be used to show differences in proliferation and to quantify cell death under different experimental conditions, such as stimulation with death‐inducing agents or exposure to genotoxic agents. So, it is possible to set this protocol up for multiple conditions, where untreated cells are considered as 100% and used as controls to obtain relative values of cell viability, provided that the same number of starting cells are used per condition. This assay is easy and reproducible, and does not require a standard curve. The cells isolated in Basic Protocol 1 are sub‐cultured in a 6‐well plate, and the count of viable cells is performed in a 96‐well plate. One 6‐well plate is required to be prepared per experimental condition to be compared.

*CAUTION*: Crystal violet is a toxic dye and requires disposal according to Environmental Health and Safety hazardous waste regulations.

### Materials

FSK cells (from Basic Protocol 1)
Complete fibroblasts cell culture medium (FBM; see recipe)
Dulbecco's phosphate‐buffered saline (DPBS; Millipore, cat. no. D8537)
0.5% crystal violet (see recipe)
100% methanol

Laminar flow biosafety hood/cabinet
Inverted microscope
50‐ml conical tubes (e.g., BD Falcon)
6‐ and 96‐well plates (Thermo Fisher Scientific, cat. no. 145380; Thermo Fisher Scientific, cat. no. 475094), gelatin coated as described in Strategic Planning
Orbital shaker
Clean paper towels
Aluminum foil
Plate reader or spectrophotometer
37°C humidified 5% carbon dioxide incubator

Additional reagents and equipment for counting viable cells with a hemocytometer and trypan blue (see Current Protocols article: Phelan, 2007)

1 From Basic Protocol 1, step 28, count 6 × 10^5^ viable cells using a hemocytometer (see Current Protocols article: Phelan, 2007) and transfer to a 50‐ml conical tube.
2 Resuspend the cells in 18 ml of complete FBM and aliquot 3 ml/well in a 6‐well, gelatin‐coated plate. Incubate at 37°C in an atmosphere of 5% CO_2_ and let the cells adhere for 24 hr, until they reach 80% confluence.
3 Remove the medium and wash the cells three times, each time with 3 ml DPBS/well.
4 Gently add 500 μl of 0.5% crystal violet.
5 Cover the plate with aluminum foil. Incubate for 30 min at room temperature. During the incubation time, gently swirl the plate using an orbital shaker.
6 Remove the staining solution and dispose of it as toxic waste.
7 With a pipette, wash the plate several times with tap water (3 ml/well) until free color is no longer visible.
8 Let the plate to dry up‐side‐down over a paper towel for 10‐15 min at room temperature.
9 Add 500 μl of 100% methanol to solubilize the crystal violet color absorbed by viable cells.
10 Agitate the plate for 1 hr on an orbital shaker at room temperature.
11 From each well of the 6‐well plate, transfer 100 μl into a 96‐well plate to allow reading with a spectrophotometer. Use 100 μl of methanol 100% as blank.
12 Read absorbance λ = 570 with a plate reader or a spectrophotometer. For relative quantification, the average OD value obtained for control cells is set to 100%, and experimental values are expressed as percentage of control (see Fig. 5). The mean and the standard error of the mean must be calculated for at least three independent experiments (in addition to multiple technical replicates per plate). Note that for each experiment, the same number of cells should be used for comparable results. The OD value for the blank (background) should be similar to the OD value of an empty well to avoid interference (refer to Critical Parameters and Troubleshooting, discussion of Basic Protocol 2).
  The absorbance is directly proportional to the number of viable cells.

## Basic Protocol 3 TRANSMISSION ELECTRON MICROSCOPY TO STUDY CELLULAR DAMAGE AND MITOCHONDRIAL DYSFUNCTION

**Figure 6:**
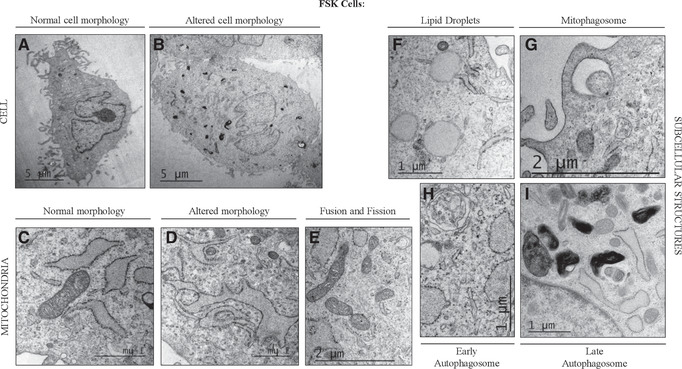
Representative electron microscopy micrographs of human primary foreskin fibroblasts (FSK). (**A**) Representative electron micrograph of FSK cells under physiological conditions and (**B**) in the presence of altered intracellular level of formaldehyde. Scale bar = 5 µm. (**C**) Representative electron microscopy micrograph of mitochondria in FSK cells under physiological conditions and (**D**) in the presence of altered intracellular level of formaldehyde. Scale bar = 1 µm. (**E**) Representative electron microscopy micrograph showing mitochondrial fusion and fission events in FSK cells. Scale bar = 2 µm. (**F‐I**) Representative electron microscopy micrographs of subcellular organelles in FSK cells, like (**F**) lipid droplets and components driving autophagy, (**G**) mitophagosome, (**H**) early autophagosome, and (**I**) late autophagosome. (**F‐H‐I**) Scale bar = 1 µm; (**G**) Scale bar = 2 µm.

Transmission electron microscopy (TEM) is a powerful technology in cell biology that allows the visualization of cells, from the ultrastructure to the molecular level (Koster & Klumperman, 2003), representing a useful approach to improve our understanding of cellular functions. All cellular components, including organelles and membrane systems, as well as specialized structures, have been characterized by TEM. Here, we describe a relatively simple step‐by step protocol, where cells are collected by trypsinization, fixed with glutaraldehyde, and dehydrated in a series of increasing concentrations of ethanol. In this way, the cells can ultimately be embedded in resin and prepared for TEM. This protocol can be used to compare cells grown under different conditions, for which TEM can be used to study morphology, cellular damage, and mitochondrial dynamics (Fig. 6). The following procedure applies to adherent cell cultures. The protocol includes toxic/hazardous reagents that require disposal according to Environmental Health and Safety hazardous waste regulations. They are labeled as such throughout the protocol.

### Materials

FSK cells (from Basic Protocol 1, step 28) in 75T cell culture flasks, cap vented (Sigma, cat. no. CLS3290), grown under experimental conditions of interest
Complete fibroblast cell culture medium (FBM; see recipe)
Dulbecco's phosphate‐buffered saline (DPBS; Millipore, cat. no. D8537)
0.05% trypsin‐EDTA (w/v) (Gibco, cat. no. T4049)
Fixative solution (see recipe) (HAZARDOUS)
0.1 M cacodylate buffer (see recipe) (HAZARDOUS)
Post‐fixative solution (see recipe) (HAZARDOUS)
EM‐grade H_2_O (Electron Microscopy Sciences
Deionized H_2_O (Fisher Scientific, cat. no. 50‐296‐94)
Ethanol series made with EM grade H_2_O and 100% ethanol (200 Proof, Decon, cat. no. 2716): 50%, 70%, 80%, 90%, 95%
Propylene oxide (Electron Microscopy Sciences, cat. no. 20401) (HAZARDOUS)
*Propylene oxide is very volatile and toxic*
EPON embedding resin (see recipe) (HAZARDOUS)
Devcon 5 min epoxy clear (Devcon, cat. no. 14200)
Chloroform (Sigma‐Aldrich, cat. no. 2432‐25 ml) (HAZARDOUS)
2% uranyl acetate (see recipe) (HAZARDOUS)
Lead stain (see recipe) (HAZARDOUS)
Sodium hydroxide (NaOH) pellets (HAZARDOUS)

Laminar flow biosafety hood/cabinet
37°C humidified 5% carbon dioxide incubator
T‐75 cell culture flasks, vented cap (Sigma, cat. no. CLS3290), gelatin coated as described in Strategic Planning
1.5‐ml microcentrifuge tubes
Microcentrifuge
55°‐60°C oven
Coated razor blades (Electron Microscopy Sciences, cat. no. 100491‐874)
Hacksaw Electron Microscopy Sciences, cat. no. 62091‐22‐0.50)
Wooden dowel, ¼ in.
Leica ultramicrotome
Glass knife box (Electron Microscopy Sciences, cat. no. 72020‐02)
Glass knife strips, 6.4 mm × 25 mm × 400 mm (Electron Microscopy Sciences, cat. no. 71012)
Diamond knife (Electron Microscopy Sciences, cat. no. 50‐363‐482)
Whatman filter paper (Millipore Sigma, cat. no. WHA1006070)
Electron microscopy grids, 300 mesh (Electron Microscopy Sciences, cat. no. EMS300‐Cu)
Parafilm
Glass petri dishes (Corning, cat. no. CLS7016560)
20‐ml plastic disposable syringe
0.2‐μm syringe filters
Dark box
Beakers, 50 and 100 ml (Corning, cat. no. 1000‐50; Corning, cat. no. 1000‐100)
Plastic transfer pipettes
Reverse forceps (Electron Microscopy Sciences, cat. no. 0202‐N0‐PO)
Grid box (Electron Microscopy Sciences, cat. no. 71150)
Whatman filter paper (Millipore Sigma, cat. no. WHA1006070)
Perfect loop (Electron Microscopy Sciences, cat. no. 70939)
BEEM embedding capsule size 00 (Electron Microscopy Sciences, cat. no. 70000‐B)
Zerostat Static gun (Electron Microscopy Sciences, cat. no. 60610)
Electron Microscope (FEI Tecnai 12 or Philips CM 12)

*CAUTION*: Care should be taken with reagents marked “HAZARDOUS” in the list above.

### Fixation

1 From Basic Protocol 1, step 28, 2 × 10^6^ viable cells in each of the required number of gelatin‐coated T‐75 flasks, with vented cap in 25 ml complete FBM, according to the experimental conditions of interest. Incubate the cells at 37°C in an atmosphere of 5% CO_2_ for the desired incubation time.
  Count cells using a hemocytometer and trypan blue exclusion (see Current Protocols article: Phelan, 2007).
2 After incubation, remove the medium from each the T‐75 coated‐flasks, wash the cells twice, each time with 25 ml DPBS, add 1 ml of 0.05% trypsin‐EDTA, and incubate at 37°C in an atmosphere of 5% CO_2_ for 3‐5 min to dislodge the cells. Stop the reaction with 10 ml of complete FBM.
3 Count the cells with a hemocytometer and collect 3 × 10^6^ FSK cells in a 1.5‐ml microcentrifuge tube. Microcentrifuge 5 min at 500 × *g*, room temperature.
4 Discard the supernatant, wash the cell pellet once with DPBS, and microcentrifuge 5 min at 500 × *g*, room temperature.
5 Discard the liquid and add 1 ml of fixative solution.
6 Incubate at room temperature for 1 hr, and gently swirl the tube every 15 min to resuspend the cell pellet and allow the cells to be homogeneously fixed.
7 Centrifuge the sample 5 min at 500 × *g*, room temperature.
8 Discard the liquid and wash the cell pellet three times, each time with 1 ml of 0.1 M cacodylate buffer.

### Post‐fixation

9 Add 1 ml of post‐fixative solution.
10 Incubate in the dark at room temperature for 15‐20 min
  The cell pellet will turn black.
11 Microcentrifuge the cell pellet 5 min at 500 × *g*, room temperature.
12 Discard the liquid and wash the cell pellet three times, each with 1 ml EM‐grade H_2_O.
  The sample can be stored at 4°C overnight before continuing with the dehydration process.

### Dehydration

The dehydration step is very important. All the water needs to be gradually removed from the cells in order to allow proper embedding in the resin, preventing subcellular morphology alteration.

13 Microcentrifuge the cell pellet for 5 min at 500 × *g*, room temperature.
14 Discard the liquid and start dehydration: add 1 ml of 50% ethanol and incubate for 5 min at room temperature.
15 Repeat steps 13 and 14.
16 Microcentrifuge 5 min at 500 × *g*, room temperature, discard the liquid, and add 1 ml of 70% ethanol.
17 Incubate 5 min at room temperature and repeat step 16.
18 Microcentrifuge 5 min at 500 × *g*, room temperature, discard the liquid, and add 1 ml of 80% ethanol.
19 Repeat step 18.
20 Microcentrifuge at 5 min at 500 × *g*, room temperature, discard the liquid, and add 1 ml of 90% ethanol.
21 Repeat step 20.
22 Microcentrifuge for 5 min at 500 × *g*, room temperature, discard the liquid, and add 1 ml of 95% ethanol.
23 Repeat step 22.
24 Microcentrifuge 5 min at 500 × *g*, at room temperature, discard the liquid, and add 1 ml of 100% ethanol.
25 Repeat step 24 twice.

### Infiltration of cells

26 Centrifuge 5 min at 500 × *g*, room temperature, and, in a laminar flow hood, add 1 ml of propylene oxide.
27 Incubate for 5 min at room temperature in a laminar flow hood.
28 Repeat steps 26 and 27.
29 Microcentrifuge 5 min at 500 × *g* room temperature, and remove the liquid.

### Embedding

**Figure 7:**
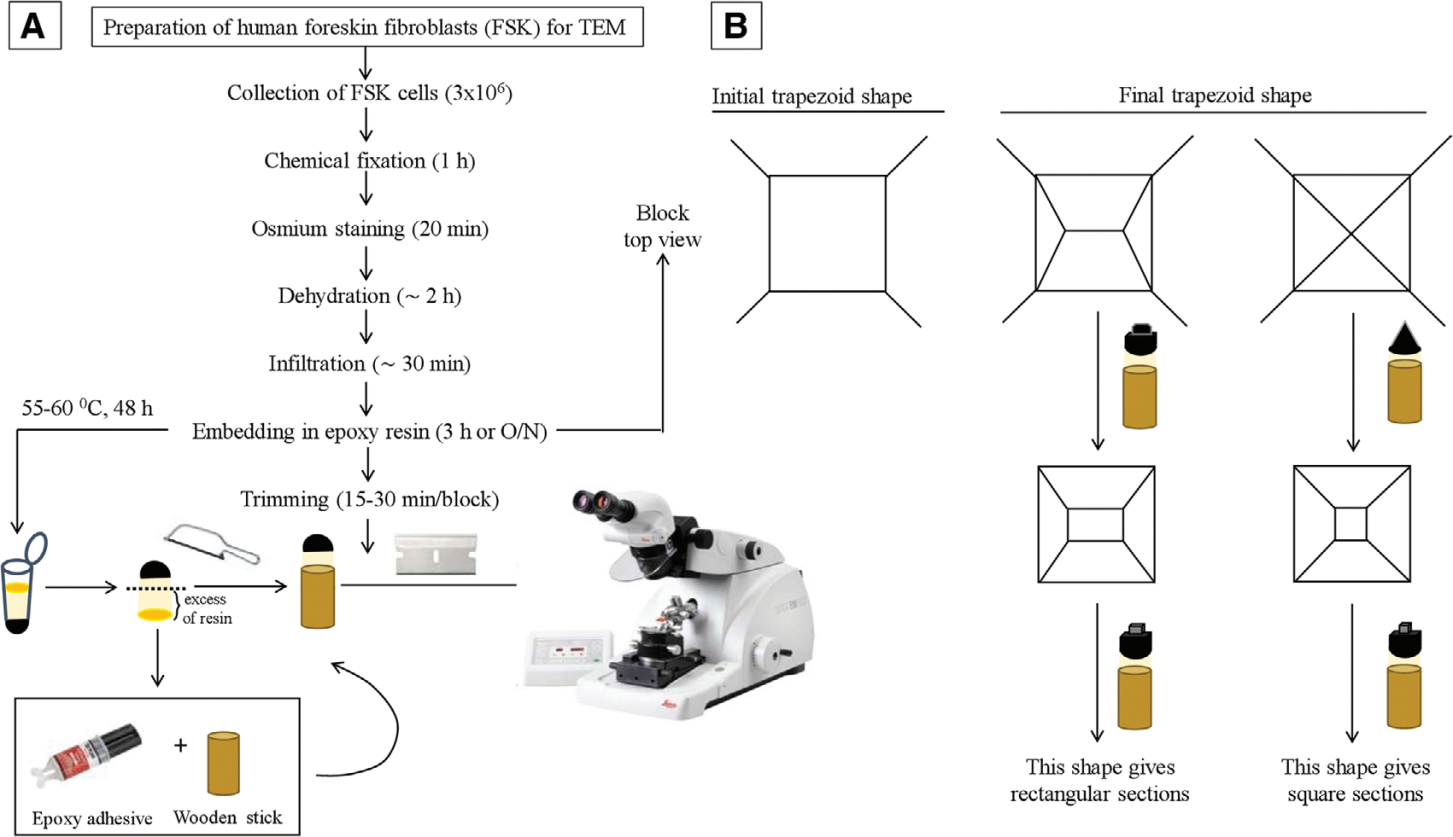
Preparation of human primary foreskin fibroblasts (FSK) for transmission electron microscopy (TEM) analysis (Basic Protocol 3). (**A**) Flowchart depicting our strategy for embedding FSK cells in EPON resin and (**B**) trimming for ultramicrotome ultra‐thin sectioning. After 48 hr at 55°C‐60°C, the resin‐embedded sample is removed from the microcentrifuge tube with a razor blade. The excess resin is cut with a hacksaw and mounted on a wooden stick with epoxy adhesive. The mounted sample can be placed on the ultramicrotome holder and trimmed. The tip of the trapezoid can be a point, giving square sections, or a ridge, resulting in rectangular sections. At this point, the sample is ready for the preparation of the sections for TEM.

30 Add 1 ml of 1:1 EPON embedding resin/propylene oxide and incubate at room temperature for 1 hr in a laminar flow hood.
31 Centrifuge 5 min at 500 × *g*, room temperature.
32 Discard the supernatant.
33 Add 1 ml 3:1 EPON/propylene oxide and incubate at room temperature for 1 hr in a laminar flow hood
  The sample can be stored at 4°C over the weekend before continuing with the next step.
34 Centrifuge 5 min at 500 × *g*, room temperature.
35 Discard the supernatant.
36 Add 1 ml EPON and incubate at room temperature for 1 hr in a laminar flow hood.
37 Centrifuge 5 min at 500 × *g*, room temperature.
38 Repeat steps 36 and 37.
39 Label the microcentrifuge tube with the date and the name of the sample. Discard supernatant and replace with 1 ml of fresh EPON.
40 Place the sample in an oven at 55°‐60°C for 48 hr to allow polymerization of the resin.
41 With a razor blade, cut the microcentrifuge tube to obtain the resin block with the black cell pellet (Fig. 7A).

42 Cut off the excess resin in excess with a hacksaw and glue with Devcon 5 min epoxy clear onto a ¼‐in. wooden dowel. See Figure 7 as a reference.
  Removing resin in excess should leave behind the sample plus no more than 0.5 cm of resin.
43 Let dry for 15‐30 min at room temperature.

### Trimming and thin sectioning

**Figure 8:**
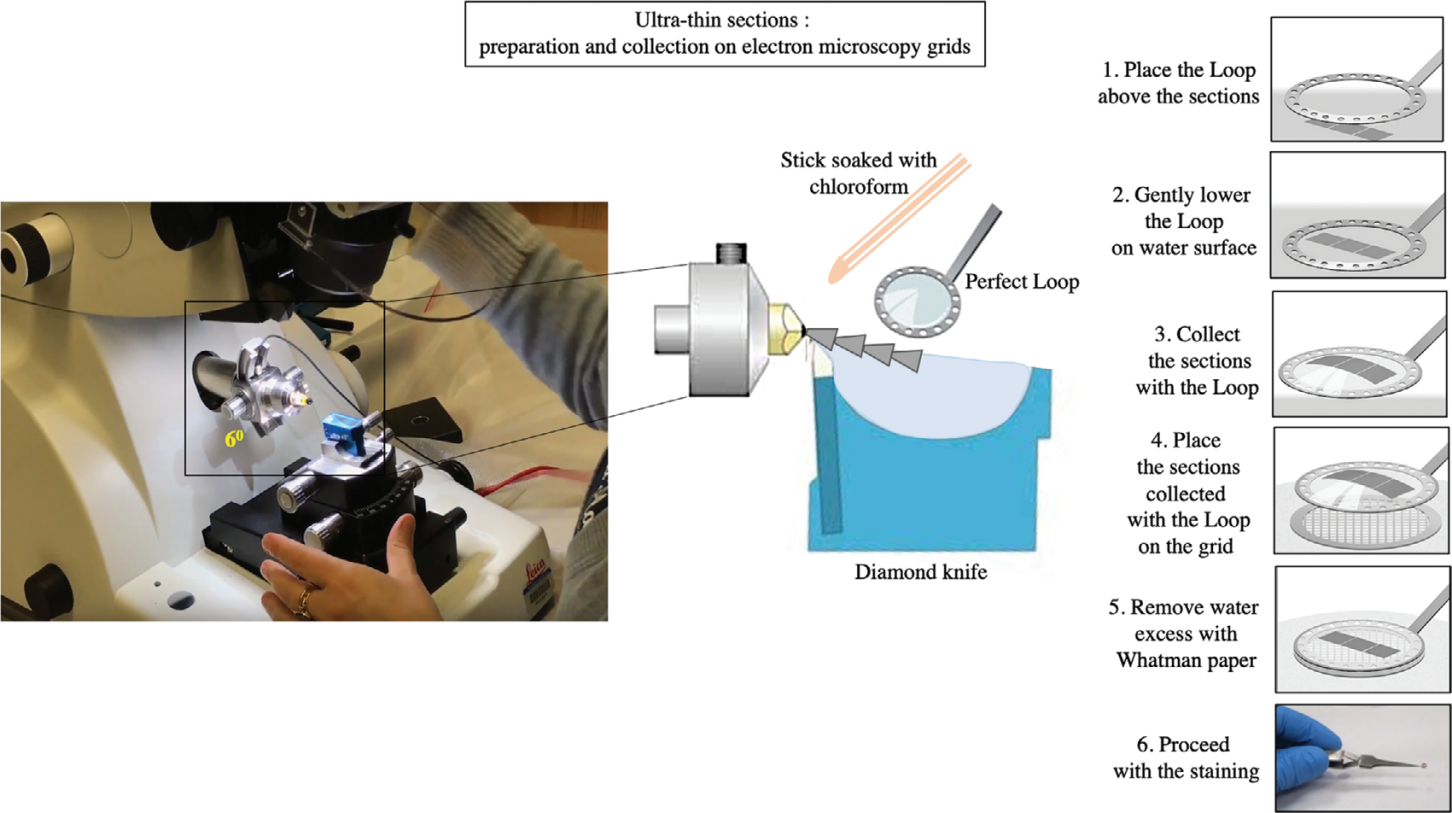
Preparation and collection of ultra‐thin sections on electron microscopy (EM) grids. Ultra‐thin sections of embedded human primary foreskin fibroblasts are prepared with a Leica ultramicrotome using a diamond knife. The sections are collected with the perfect loop and transferred to EM grids. Excess water is removed with Whatman paper.

44 In an ultramicrotome, place the block into the specimen holder and tighten the clamping screw. The arm needs to be in the locked position. Make sure that the block is firmly fastened into the holder, as loose blocks can interfere with sectioning.
45 Trim the block to a truncated pyramid (Fig. 7B), with the base section area being as small as possible. This helps to prepare good sections later. Using a clean razor blade and looking down the binoculars of the inverted microscope, remove the excess resin from each of the four sides of the block to obtain a truncated pyramid (Fig. 7B).
46 Use a glass knife to trim the front surface of the block before you start cutting the specimen. At this stage, cut semi‐thin sections of about 500 nm to get access to the specimen.
47 Smooth the surface with a glass knife (examine the knife in the ultramicrotome using the black light to ensure that the edge is sharp and dust‐free).
48 Replace the glass knife with a diamond knife (see Fig. 8). Fill the diamond knife holder with EM‐grade H_2_O, and check that the holder is tightly fastened and at a clearance angle of 6°.

49 Advance the knife/knife holder by hand to within ∼1 mm of the block and clamp the holder in position.
50 With the specimen arm in the upper, locked position, adjust the position of the block so that the top of the block is positioned just below the knife edge.
  This sets the end of the cutting stroke (when the motor cuts‐in again to initiate another cutting cycle) and, by default, also sets the beginning of the free‐fall cutting stroke (this is ∼4 mm above the knife edge).
51 Advance the knife stage using the wheel at the front of the microtome until only a thin slit of reflected light is visible on the specimen face. This slit of light should be of the same thickness along the whole face of the block. This means that the block face and the knife edge are parallel in the left‐right direction. Proceed with any necessary rotational or lateral adjustments to make this slit of light parallel.
52 Carefully move the block up and down in front of the knife and watch the slit of light to see if it changes in thickness.
53 Align the block face to the knife.
54 Advance the knife until the thin slit of light begins to appear yellow and/or disappears. At this stage, the knife is approximately 1 μm from the specimen face.
55 Fill the knife boat with filtered distilled H_2_O until a silvered reflection is visible off the surface of the liquid.
56 Start cutting individual sections by hand and then activate the motor to begin automatic sectioning in a ribbon on the surface of the water.
57 Adjust the section thickness settings to obtain pale gold or silver sections.
  In conventional TEM, sections need to be in the range of 50‐70 nm of thickness to obtain sharply focused images at high magnification (>30,000×). Semi‐thin sections allow samples to be screened to select specific areas for thin sectioning. Semi‐thin sections are cut between 0.5 and 1 μm from trimmed blocks and viewed by bright‐field microscopy. Various cationic dyes, including crystal violet, toluidine blue, and methylene blue, can be used for contrast.

### Picking up ultra‐thin sections on a grid

58 Expand the sections with chloroform (Fig. 8). For this, use either a thin, wedge‐shaped piece of filter paper or a cotton swab soaked in the chloroform, and gently wave it over the sections. Be careful not to over‐expand the sections, as this can lead to damage (cracks and /or loss of material from the section).
59 Collect the sections onto a grid by carefully lowering the grid, dull‐side‐downwards, onto the sections.
  An average of 3‐10 sections can be collected onto each grid.
60 Place the grid onto a piece of filter paper with the sections facing upwards. The water will be absorbed through the grid mesh and the sections will become visible on the grid.
  An alternative method of collecting sections is by putting the grid under the liquid in the knife boat and then coming up under the sections with the grid.
61 Let the grids completely dry on filter paper before proceeding with the staining.

### Lead stain (Sato's staining)

**Figure 9:**
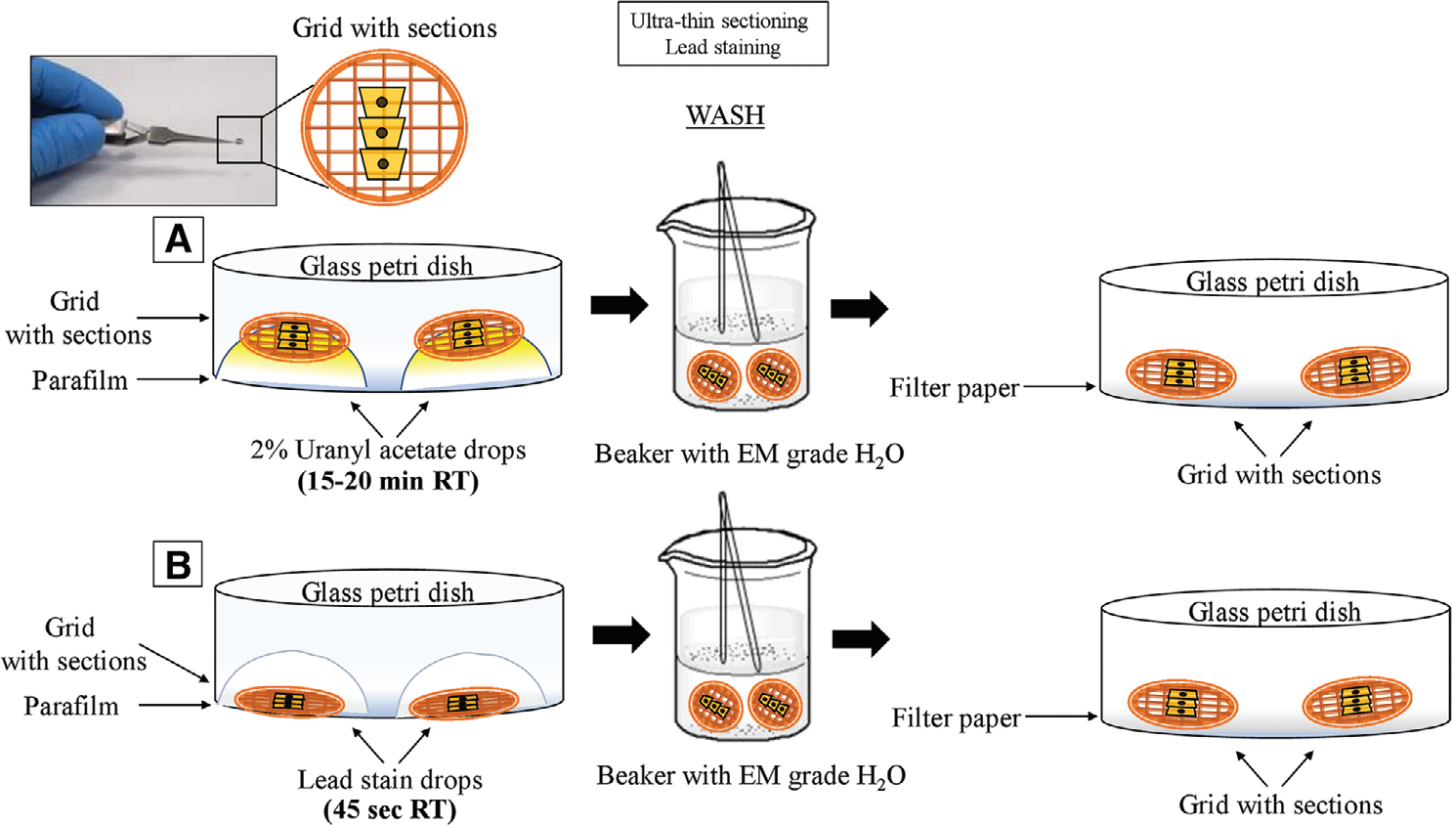
Lead staining of ultra‐thin sections for electron microscopy visualization. Ultra‐thin sections are stained with uranyl acetate (**A**) and lead (**B**) for electron microscopy analysis. The grids are allowed to dry, and excess water is removed with Whatman paper.

62 Cut the parafilm into 10 × 10 cm size pieces and place in a glass petri dish. Do not touch the surface of the parafilm with your hands prior to staining (Fig. 9A).

63 Take 1 ml of 2% uranyl acetate in a 1.5‐ml microcentrifuge tube and centrifuge 5 min at 13,000 × *g*, room temperature.
64 Transfer to a 1‐ml syringe attached to a 0.2‐µm syringe filter.
65 Discard the first drop and put a series of drops onto the Parafilm according to the number of grids to stain.
66 Lower the grids onto the surface of the uranyl acetate drops (the uranyl acetate impurities are on the bottom of the drops).
67 Cover with a dark box to protect from light.
68 Incubate at room temperature for 15‐20 min.
69 Prepare a couple of beakers (50 or 100 ml) with distilled H_2_O.
70 After incubation, place the grids into the beakers and wash using a plastic pipette.
71 Collect the grids with reverse forceps. Wash with some additional drops of distilled H_2_O using a syringe with a filter.
72 Blot from the side with filter paper and lay the forceps, holding the grids, in a covered petri dish to dry. The drying can be done on a clean filter paper in a covered glass petri dish. To complete drying, place the grids into the grid box.
73 Prepare lead stain as described in Reagents and Solutions.
74 Take an aliquot of the lead stain solution (1 ml) in a microcentrifuge tube and spin it down in a tabletop microcentrifuge for 2‐3 min, at maximum speed.
  The remaining solution is stored in the syringe at 4°C. The use of the syringe helps to minimize/prevent the exposure of the solution to the air that may cause the formation of precipitates.
75 Cut some parafilm and place it in a glass petri dish. Add some pellets of NaOH (3‐4 grains; 1 grain size = 0.5 × 0.3 mm).
76 Bleed off the first drop and put a series of drops on the parafilm, according to the number of grids to stain (Fig. 9B).
77 Take the grids and place them on the bottom of the drops by perpendicular immersion. Do not breathe over the lead stain, as this will cause CO_2_ contamination and subsequently, background.
78 Incubate for 45 s at room temperature and immediately place the grids into beakers with distilled H_2_O.
79 Wash the grids with a plastic pipette.
80 Remove the grids with the reverse forceps and wash with some additional drops of distilled H_2_O using a syringe with filter.
81 Blot from the side with filter paper and lay forceps holding grids in a covered petri dish to dry. To complete drying, put the grids into the grid box. Alternatively, the dry treatment can be done on a clean filter paper in a covered glass petri dish.
  Grids are now ready for electron microscopy analysis.

## REAGENTS AND SOLUTIONS

### Cacodylate buffer 0.1 M (for 500 ml)

10.7 g cacodylic acid, sodium salt, trihydrate (Electron Microscopy Sciences, cat. no. 12310) (HAZARDOUS)
450 ml EM‐grade H_2_O (Electron Microscopy Sciences deionized water, Fisher Scientific, cat. no. 50‐296‐94)
Adjust pH to 7.2 with 12 M HCl (see above)
Bring volume to 500 ml with EM‐grade H_2_O (Electron Microscopy Sciences deionized water, Fisher Scientific, cat. no. 50‐296‐94)
Store up to 6 months at 4°C
  CAUTION: Cacodylate buffer is highly toxic and requires disposal according to Environmental Health and Safety hazardous waste regulations.

### Cacodylate buffer 0.2 M (for 500 ml)

21.4 g cacodylic acid, sodium salt, trihydrate (Electron Microscopy Sciences, cat. no. 12310) (HAZARDOUS)
450 ml EM‐grade H_2_O (Electron Microscopy Sciences deionized water, Fisher Scientific, cat. no. 50‐296‐94)
Adjust the pH to 7.2 with 1 M HCl
Bring volume to 500 ml with EM grade H_2_O (Electron Microscopy Sciences deionized water, Fisher Scientific, cat. no. 50‐296‐94)
Store up to 6 months at 4°C

### Complete fibroblast cell culture medium (FBM, 560 ml)

500 ml Dulbecco's modified Eagle's medium (DMEM, Gibco, cat. no. 11885‐084)
50 ml fetal bovine serum (FBS, HyClone, cat. no. SH30396.03)
5 ml 100× non‐essential amino acids (Gibco, cat. no. 11140‐0500
5 ml 100× penicillin/streptomycin/amphotericin B solution (Thermo Fisher Scientific, cat. no. 15240062)
  The medium can be stored up to 1 month at 4°C. Avoid heating to reduce degradation of glutamine and growth factors

### Crystal violet 0.5% staining solution (for 100 ml)

500 mg crystal violet powder (Sigma, cat. no. C0775) (HAZARDOUS)
100 ml 50% methanol (50 ml dH_2_O + 50 ml 100% methanol)
  CAUTION: This solution is highly toxic and requires disposal according to the Environmental Health and Safety hazardous waste regulations.
  NOTE: The crystal violet solution can be stored in the dark at room temperature for up to 6 months.

### Embedding resin (EPON)

50 g EMBed 812 (a liquid: take 25 ml twice and weigh up to 50 g) (Electron Microscopy Sciences, cat. no. 14120)
25 g DDSA (a liquid: take about 28 ml) (Electron Microscopy Sciences, cat. no. 13710)
26.9 g NMA (a liquid: take about 25 ml) (Electron Microscopy Sciences, cat. no. 19000)
50 drops DMP‐30 (Electron Microscopy Sciences, cat. no. 13600) [use glass Pasteur pipette; mix with a wooden stirrer (Electron Microscopy Sciences, cat. no. 72300), avoiding air bubbles]
Pull up into 25‐ml syringes, cap, and store in the freezer up to 6 months. Fresh resin is bright orange and becomes yellow after freezing. To thaw, place in a plastic zip‐lock bag with desiccant and leave at room temperature prior embedding. Refreeze remaining resin.
  CAUTION: EPON resin is a teratogen and also is mutagenic. The material needs to be handled with care in a laminar flow hood and requires disposal according to Environmental Health and Safety hazardous waste regulations.

### Fixative solution (for 25 ml)

12.5 ml 0.2 M sodium cacodylate buffer, pH 7.2 (HAZARDOUS)
2.5 ml 25% EM‐grade glutaraldehyde (Electron Microscopy Sciences, cat. no. 1622) (HAZARDOUS)
20 ml EM‐grade H_2_O (Electron Microscopy Sciences deionized water, Fisher Scientific, cat. no. 50‐296‐94)
Adjust pH up to 7.2 with 1 N NaOH
Bring final volume to 25 ml
  NOTE: The fixative needs always to be freshly prepared prior to use. This solution is highly toxic and requires disposal according to the Environmental Health and Safety hazardous waste regulations.

### Lead stain

1 g lead nitrate (Electron Microscopy Sciences, cat. no. 17900)
1 g lead citrate (Electron Microscopy Sciences, cat. no. 17810)
1 g lead acetate (Electron Microscopy Sciences, cat. no. 17600)
2 g sodium citrate (Electron Microscopy Sciences, cat. no. 21140)
82 ml boiled and then cooled down distilled water
18 ml 1 N NaOH
Aliquot the lead stain into five new 25‐ml syringes (25 ml per syringe), avoiding any residual airspace, and seal the ends very well with parafilm. Store up to 6 months at 4°C.

### Post‐fixative solution (for 1 ml)

125 µl 4% osmium tetroxide (Electron Microscopy Sciences, cat. no. 19170) (HAZARDOUS)
875 µl 0.1 M sodium cacodylate buffer, pH 7.2 (HAZARDOUS)
  CAUTION: Osmium is particularly dangerous. The crystals, liquid, and vapors all are hazardous. The vapors can fix the cornea and lens of the eye and both vapors and liquid are absorbed rapidly through the skin and act as a nerve gas (it was used as such in the first world war). Osmium can be used only with proper ventilation (e.g., fume hood). Report any and all accidents immediately. Osmium and sodium cacodylate buffer are highly toxic and always must be prepared prior to use in a laminar flow hood. Disposal must follow Environmental Health and Safety hazardous waste regulations.

### Uranyl acetate

0.5 g uranyl acetate (Electron Microscopy Sciences, cat. no. 22400)
25 ml 50% ethanol
Prepare 25 ml of 2% uranyl acetate (UA) in 50% ethanol (first prepare 50% ethanol and then add the UA). Store at room temperature in the dark or in a dark bottle for up to 1 month.
  CAUTION: The solution is light sensitive. Though the external radiological and chemical danger of UA is relatively low, it is ordered as a powder and reconstituted in the lab, thus increasing the potential for exposure to inhalation or ingestion. Follow Laboratory Safety Guidelines to work with uranium staining. Due to the toxicity hazard, uranium staining solutions may not be poured down a sink for disposal. Liquids should be collected into radioactive liquid waste containers with absorbent. Contact Radiation Safety Service for waste pickup according to Environmental Health and Safety hazardous waste regulations.

## COMMENTARY

### Background Information

The skin represents an important source of cells that can be isolated and used in different areas of research (Rognoni & Watt, 2018). The use of cutaneous cells represents a less invasive source of human cells for laboratory applications, especially if we consider that, for instance, after surgery, some tissues are discarded. Fibroblasts are mesenchymal cells and the most abundant cell type in preputial skin. These cells produce growth factors and ECM components, and express receptors for hormones and neurotransmitters (Kendall & Feghali‐Bostwick, 2014). Several studies have demonstrated the resourcefulness of human foreskin fibroblasts (FSK) in many research areas and how they offer an advantageous model to study inflammation and complex diseases, and even to support the growth of human embryonic stem cells or other cell types in culture (Ichim, O'Heeron, & Kesari, 2018). FSK cells also have been employed in epigenetic studies (Albrengues et al., 2015; Neary, Watson, & Baugh, 2015). Overall, the study of these cells has significantly contributed to the advancement of our knowledge in cell biology and disease pathogenesis. Consequently, the preparation of primary cultures from human foreskin tissue and their subsequent study may provide a valuable approach for analyzing the consequences of altered physiological conditions at the cellular and sub‐cellular levels. The viability of these cells, under various conditions, can be easily measured with crystal violet staining, one of the most common and versatile methods used to evaluate cell wellbeing. Crystal violet is a triarylmethane cationic dye that is efficiently sequestered by viable cells. Dead adherent cells detach from cell culture plates and are removed during washing steps, and thus do not contribute to the signal measured in the spectrophotometer. The staining is quick, simple, and sensitive for screening relative cell viability under diverse stimulation or inhibition conditions.

FSK cells may undergo morphological changes and metabolic adaptations in response to a variety of extracellular and intracellular signals. This flexibility is safeguarded by the mitochondria, which rapidly divide and fuse in a dynamic network, thus providing support to the cells (Scott & Youle, 2010). Fusion and fission are essential mitochondrial activities, as evidenced by the fact that many human diseases are caused by mutations in genes that control these processes (Archer, 2013). Under normal conditions, accumulation of dysfunctional mitochondria is prevented by mitophagy; this was first described in 1966 using the TEM approach (De Duve & Wattiaux, 1966). In the last decades, improvements on classical techniques and instrumentation for TEM have made possible the magnification and observation of cells and organelles at greater resolution, with extraordinary detail and accuracy, including mitochondria (Rybka et al., 2019). The shape and number of mitochondria correlate with the physiological state of the cell (Scott & Youle, 2010), and the topology of outer and inner mitochondrial membranes indicates the balance between fusion and fission events. Mitochondrial ultrastructure is best revealed directly under the high resolution afforded by TEM; this has been successfully used to distinguish several mitochondrial features (Figs. 6 and 2; (Eustaquio et al., 2018; Nadalutti et al., 2020; Rybka et al., 2019). It is well established that, e.g., under oxidative stress conditions, mitochondrial outer and inner membranes reorganize; the mitochondrial membrane potential is lost, and mitochondria undergo fragmentation to allow the cellular clearance of damaged organelles through mitophagy (Luciani et al., 2020; Perrotta & Aquila, 2015). These parameters, as well as other specific features of mitochondria (Fig. 2), can be quantitatively and qualitatively measured by TEM, which is why we have focused here on preparing samples to be studied using this technique to study mitochondrial dynamics. From more than 100 EM micrographs, individual cells and mitochondria can be analyzed. Knowledge of the changes that are occurring in mitochondria may provide important insight into the key mechanisms underlying various physiological and pathological conditions.

### Critical Parameters and Troubleshooting

#### Basic Protocol 1

During isolation of human primary cells, contamination with other cell types may occur. To prevent and limit contamination with other cell types, it is recommended to wash the tissue specimen obtained from the clinic several times in balanced salt solution with 1% added antibiotics and antimycotics before cell isolation.

Primary cells are very sensitive and should be gently handled. Any shear force can represent an exogenous source of cellular damage. In addition, attention should be paid to avoid that primary cells become too confluent (80%‐100%), because this may cause cell senescence and induce altered morphology. Cells must be passaged once or twice per week, avoiding long trypsin digestion that can be harmful. If the cells do not grow, the amount of fetal bovine serum can be increased up to 20%, or trypsin should be added in a smaller volume and for a shorter time.

If mycoplasma infection is suspected, the plate with the cells should be isolated immediately and tested for mycoplasma infection. If the test turns positive, the incubator must be carefully cleaned.

We recommend the use of FSK cells up to passage 6 to limit genetic drift and functional changes. Over time, cells can experience senescence or modify protein expression. Even though cells continue to proliferate, they may not be suitable for experiments.

#### Basic Protocol 2

Check to verify that you have the proper filter for the microplate reader. High OD background values may indicate contamination of the plate and/or the solutions. New plates should be used and solutions must be freshly prepared. If the signal is too high, the sample must be diluted. In fact, too‐high OD sample values correlate with too many cells. In this case, the cells should be diluted (for example 1:2 or 1:10) until the readings are not saturated. If the cells detach from the plate, the washes may be too strong. To avoid detaching the cells from the plate, pipetting always must be done on the side of the plate.

#### Basic Protocol 3

Poor fixation is evaluated by observing the mitochondria for enlarged, altered cristae organization and distribution, as well as for the presence of extracellular spaces. The pH of the buffer and osmolarity are crucial for the success of the protocol. Always check that the pH is correct. Prepare a fresh batch of glutaraldehyde for every procedure. Observe the recommended fixative incubation time, because poor fixation can result from too short an incubation time with the fixatives. It is possible to increase the incubation time up to 2 hr. Do not exceed this time, to avoid morphological changes in the cells.

Soft blocks indicate modest polymerization. Check the expiration date of all the components used in the preparation of the resin and thoroughly mix them. Manually invert the tube to prevent the introduction of air bubbles during the preparation of the resin. Replace accelerator every 6 months and prepare fresh resin. Brittle blocks are caused by poor dehydration (analytical‐grade solvents are not used; incorrect concentrations of solvents are used; serial ethanol dehydration times are too short). Control expiration date of accelerator and check the concentration added during the preparation of the resin, because this should correspond to the recommended volume. If the sections will not cut, make the block face smaller, check the position of the cutting stroke, and control the knife angle, the speed of the arm‐drop, and the level of the water bath. If the sections flow over the back of the knife after cutting, check the knife angle, the speed of the arm‐drop, and the level of the water bath.

Addressing potential problems with section cuts: check the block and consider trimming it again if necessary; control the knife angle and the level of the water bath; carefully clean the diamond knife and align the block properly; and check the position of the cutting stroke. If the sections shrink, the knife may be dirty. Proceed with cleaning of the knife in correspondence to the cutting area. If possible, use dust‐negative propellant.

The presence of small electron‐dense particulate material of about 10 nm in size throughout the section is generated by the lead citrate staining. Repeat the staining procedure on unstained grids, and take care not to introduce CO_2_ from the environment.

Also use high‐quality glutaraldehyde and calibrated cacodylate buffers. Follow the recommended staining incubation time, and add 1% tannic acid after fixation for 5 min at room temperature to improve the contrast of cellular structures.

### Understanding Results

#### Basic Protocol 1

It is very important to know the morphology of primary cells (see Fig. 3) to be able to identify contaminating cells. Contamination with other cell types is frequent, and FSK cells need to be characterized for the presence of specific fibroblast markers, such as vimentin, nestin, fibroblast‐specific protein‐1, CD10, CD26, and collagen VII (Garrett et al., 2017).

#### Basic Protocol 2

We have successfully used this approach to gain insight into the in vivo cytotoxic effects of formaldehyde (FA), a normal product of cell metabolism. FA is present in the human bloodstream at a concentration of about 20‐100 μM (Burgos‐Barragan et al., 2017; Liu & Locasale, 2017). Addition of exogenous FA to cells results in the dysregulation of intracellular FA level (concentrations used, 250 and 500 μM). Exposure to 250 μM FA causes a drop in cell viability (to 47%) after 24 hr, with a partial recovery after 48 and 72 hr (Fig. 5B). FSK cells were more sensitive to 500 μM FA at each time point tested. We have provided a representative experiment, with relative OD readings and quantification in Figure 5.

#### Basic Protocol 3

We have successfully employed this approach to study cellular damage and mitochondrial dysfunction after deregulation of endogenous formaldehyde (FA) levels (Nadalutti et al., 2020) by FA exogenous addition. The cells isolated in Basic Protocol 1 have been sub‐cultured in T‐75 flasks, treated with different concentrations of FA for 24 hr, and prepared for TEM as described here. We have found that increased intracellular FA causes distinct morphological changes in FSK cells (Fig. 6A‐B), as well as mitochondrial rearrangements (Fig. 6D) and lipid droplet accumulation (Fig. 6F), as a consequence of mitochondrial dysfunction. Mitochondria are known to play a central role in the regulation of different cellular activities, from energy production to fatty acid oxidation, throughout cycles of fusion and fission. Damaged mitochondria must be selectively removed by autophagy and mitophagy (Fig. 6G‐I) to avoid cellular damage. Under pathological conditions, these organelles are programmed to undergo profound morphological modifications, and TEM has been instrumental in visualizing and characterizing these changes.

### Time Considerations

Figure 4, Figure 7, and Figure 9 define the relative time considerations for the implementation of the protocols herein described. Specifically, the isolation of the cells from the foreskin specimen requires about 3 hr; the crystal violet staining can be performed in 2 hr; embedding of the cells in the resin needs approximately 7 hr; the polymerization of the resin takes 2 days; and trimming, sectioning and staining for TEM takes 3 hr.

### Conflict of Interest Statement

The authors declare no conflict of interest.

### Author Contributions

**Cristina A. Nadalutti**: Conceptualization; data curation; formal analysis; investigation; methodology; software; validation; visualization; writing‐original draft; writing‐review & editing. **Samuel H. Wilson**: Conceptualization; data curation; formal analysis; funding acquisition; methodology; project administration; resources; software; supervision; writing‐review & editing.

## References

[cptx99-bib-0001] Acharya, P. S. , Majumdar, S. , Jacob, M. , Hayden, J. , Mrass, P. , Weninger, W. , … Puré, E. (2008). Fibroblast migration is mediated by CD44‐dependent TGFβ activation. Journal of Cell Science, 121(9), 1393. doi: 10.1242/jcs.021683. 18397995

[cptx99-bib-0002] Albrengues, J. , Bertero, T. , Grasset, E. , Bonan, S. , Maiel, M. , Bourget, I. , … Gaggioli, C. (2015). Epigenetic switch drives the conversion of fibroblasts into proinvasive cancer‐associated fibroblasts. Nature Communications, 6, 10204. doi: 10.1038/ncomms10204. PMC4682161 26667266

[cptx99-bib-0003] Archer, S. L. (2013). Mitochondrial dynamics−mitochondrial fission and fusion in human diseases. New England Journal of Medicine, 369(23), 2236–2251. doi: 10.1056/NEJMra1215233. 24304053

[cptx99-bib-0004] Burgos‐Barragan, G. , Wit, N. , Meiser, J. , Dingler, F. A. , Pietzke, M. , Mulderrig, L. , … Patel, K. J. (2017). Mammals divert endogenous genotoxic formaldehyde into one‐carbon metabolism. Nature, 548(7669), 549–554. doi: 10.1038/nature23481. 28813411 PMC5714256

[cptx99-bib-0005] De Duve, C. , & Wattiaux, R. (1966). Functions of lysosomes. Annual Review of Physiology, 28, 435–492. doi: 10.1146/annurev.ph.28.030166.002251. 5322983

[cptx99-bib-0006] Dikic, I. , & Elazar, Z. (2018). Mechanism and medical implications of mammalian autophagy. Nature Reviews Molecular Cell Biology, 19(6), 349–364. doi: 10.1038/s41580-018-0003-4. 29618831

[cptx99-bib-0007] Eustaquio, T. , Wang, C. , Dugard, C. K. , George, N. I. , Liu, F. , Slikker, W., Jr ., … Paredes, A. M. (2018). Electron microscopy techniques employed to explore mitochondrial defects in the developing rat brain following ketamine treatment. Experimental Cell Research, 373(1‐2), 164–170. doi: 10.1016/j.yexcr.2018.10.009. 30342004

[cptx99-bib-0008] Feoktistova, M. , Geserick, P. , & Leverkus, M. (2016). Crystal violet assay for determining viability of cultured cells. Cold Spring Harbor Protocols, 2016(4), pdb prot087379. doi: 10.1101/pdb.prot087379. 27037069

[cptx99-bib-0009] Freshney, R. I. (1987). Culture of animal cells: A manual of basic technique. New York: Alan R. Liss, Inc. doi: 10.1002/jctb.280450414.

[cptx99-bib-0010] Friedman, J. R. , & Nunnari, J. (2014). Mitochondrial form and function. Nature, 505(7483), 335–343. doi: 10.1038/nature12985. 24429632 PMC4075653

[cptx99-bib-0011] Garrett, S. M. , Baker Frost, D. , & Feghali‐Bostwick, C. (2017). The mighty fibroblast and its utility in scleroderma research. Journal of Scleroderma Related Disorders, 2(2), 69–134. doi: 10.5301/jsrd.5000240. PMC5736140 29270465

[cptx99-bib-0012] Ichim, T. E. , O'Heeron, P. , & Kesari, S. (2018). Fibroblasts as a practical alternative to mesenchymal stem cells. Journal of Translational Medicine, 16, 212. doi: 10.1186/s12967-018-1536-1. 30053821 PMC6064181

[cptx99-bib-0013] Jiang, P. , & Mizushima, N. (2014). Autophagy and human diseases. Cell Research, 24(1), 69–79. doi: 10.1038/cr.2013.161. 24323045 PMC3879707

[cptx99-bib-0014] Kendall, R. T. , & Feghali‐Bostwick, C. A. (2014). Fibroblasts in fibrosis: Novel roles and mediators. Frontiers in Pharmacolology, 5, 123. doi: 10.3389/fphar.2014.00123. PMC4034148 24904424

[cptx99-bib-0015] Koster, A. J. , & Klumperman, J. (2003). Electron microscopy in cell biology: Integrating structure and function. Nature Cell Biology, Suppl:SS6‐10. doi: 10.1038/nrm1194. 14587520

[cptx99-bib-0016] Liu, X. , & Locasale, J. W. (2017). A toxin that fuels metabolism. Nature, 548(7669), 533–534. doi: 10.1038/nature23541. 28813408

[cptx99-bib-0017] Luciani, A. , Schumann, A. , Berquez, M. , Chen, Z. , Nieri, D. , Failli, M. , … Devuyst, O. (2020). Impaired mitophagy links mitochondrial disease to epithelial stress in methylmalonyl‐CoA mutase deficiency. Nature Communications, 11(1), 970. doi: 10.1038/s41467-020-14729-8. PMC7033137 32080200

[cptx99-bib-0018] Nadalutti, C. A. , Stefanick, D. F. , Zhao, M. L. , Horton, J. K. , Prasad, R. , Brooks, A. M. , … Wilson, S. H. (2020). Mitochondrial dysfunction and DNA damage accompany enhanced levels of formaldehyde in cultured primary human fibroblasts. Scientific Reports, 10(1), 5575. doi: 10.1038/s41598-020-61477-2. 32221313 PMC7101401

[cptx99-bib-0019] Neary, R. , Watson, C. J. , & Baugh, J. A. (2015). Epigenetics and the overhealing wound: The role of DNA methylation in fibrosis. Fibrogenesis & Tissue Repair, 8, 18. doi: 10.1186/s13069-015-0035-8. 26435749 PMC4591063

[cptx99-bib-0020] Nunnari, J. , & Suomalainen, A. (2012). Mitochondria: In sickness and in health. Cell, 148(6), 1145–1159. doi: 10.1016/j.cell.2012.02.035. 22424226 PMC5381524

[cptx99-bib-0021] Oliveira, T. , Costa, I. , Marinho, V. , Carvalho, V. , Uchoa, K. , Ayres, C. , … Vasconcelos, D. F. P. (2018). Human foreskin fibroblasts: From waste bag to important biomedical applications. Journal of Clinical Urology, 11(6), 385–394. doi: 10.1177/2051415818761526.

[cptx99-bib-0022] Perrotta, I. , & Aquila, S. (2015). The role of oxidative stress and autophagy in atherosclerosis. Oxidative Medicine and Cellular Longevity, 2015, 130315. doi: 10.1155/2015/130315. 25866599 PMC4381688

[cptx99-bib-0023] Phelan, M. C. (2007). Techniques for mammalian cell tissue culture. Current Protocols in Toxicology, 33, A.3B.1–A.3B.18. doi: 10.1002/0471140856.txa03bs33. 20972966

[cptx99-bib-0024] Pizzino, G. , Irrera, N. , Cucinotta, M. , Pallio, G. , Mannino, F. , Arcoraci, V. , … Bitto, A. (2017). Oxidative stress: Harms and benefits for human health. Oxidative Medicine and Cellular Longevity, 2017, 8416763–8416763. doi: 10.1155/2017/8416763. 28819546 PMC5551541

[cptx99-bib-0025] Rognoni, E. , & Watt, F. M. (2018). Skin cell heterogeneity in development, wound healing, and cancer. Trends in Cell Biology, 28(9), 709–722. doi: 10.1016/j.tcb.2018.05.002. 29807713 PMC6098245

[cptx99-bib-0026] Rybka, V. , Suzuki, Y. J. , Gavrish, A. S. , Dibrova, V. A. , Gychka, S. G. , & Shults, N. V. (2019). Transmission electron microscopy study of mitochondria in aging brain synapses. Antioxidants, 8(6), 171. doi: 10.3390/antiox8060171. PMC6616891 31212589

[cptx99-bib-0027] Scott, I. , & Youle, R. J. (2010). Mitochondrial fission and fusion. Essays in Biochemistry, 47, 85–98. doi: 10.1042/bse0470085. 20533902 PMC4762097

[cptx99-bib-0028] Somuncu, O. S. , Tasli, P. N. , Sisli, H. B. , Somuncu, S. , & Sahin, F. (2015). Characterization and differentiation of stem cells isolated from human newborn foreskin tissue. Applied Biochemistry and Biotechnology, 177(5), 1040–1054. doi: 10.1007/s12010-015-1795-8. 26304127

[cptx99-bib-0029] Unger, C. , Felldin, U. , Rodin, S. , Nordenskjold, A. , Dilber, S. , & Hovatta, O. (2016). Derivation of human skin fibroblast lines for feeder cells of human embryonic stem cells. Current Protocols in Stem Cell Biology, 36, 1C.7.1–1C.7.11. doi: 10.1002/9780470151808.sc01c07s36. 26840224

[cptx99-bib-0030] Youle, R. J. , & van der Bliek, A. M. (2012). Mitochondrial fission, fusion, and stress. Science, 337(6098), 1062–1065. doi: 10.1126/science.1219855. 22936770 PMC4762028

